# G-CSF utilization rate and prescribing patterns in United States: associations between physician and patient factors and GCSF use

**DOI:** 10.1002/cam4.344

**Published:** 2014-11-20

**Authors:** Gisoo Barnes, Ashutosh Pathak, Lee Schwartzberg

**Affiliations:** 1 Teva Pharmaceuticals41 Moores Rd., Frazer, Pennsylvania, 19355; 2 The West Clinic100 N. Humphreys Blvd., Memphis, Tennessee, 38120

**Keywords:** Granulocyte-colony-stimulating factor, mylosupressive chemotherapy, neutropenia

## Abstract

Febrile neutropenia (FN) is a common complication among patients with chemotherapy-induced myelotoxicity and is associated with a number of negative outcomes including prolonged hospitalization, increased medical costs, increased risk of mortality, dose reductions, and delays. Granulocyte-colony-stimulating factor (G-CSF), granulocyte–macrophage-colony stimulating factor (GM-CSF), and pegylated G-CSF are effective at reducing risk and duration of neutropenia-related events. However, despite guidelines, the use of G-CSF and pegylated G-CSF in the United States has not been consistent and pattern of care studies have focused primarily on G-CSF. A number of studies found that G-CSF is underutilized in patients undergoing chemotherapy treatments associated with a high risk of FN, while being over utilized in patients with a low-risk FN. Wide variations in overuse, underuse, and misuse of G-CSF are associated with a number of physician and patient factors. Improved awareness of the guidelines, feedback to providers regarding proper usage, and understanding of chemotherapy regimens associated with very low risks as well as high risks (>20%) of FN is some of the approaches that could lead to improving care.

## Introduction

Neutropenia is a common complication among patients with chemotherapy-induced myelotoxicity. Severe neutropenia (SN) and febrile neutropenia (FN: neutropenia with fever) are associated with prolonged hospitalization, serious infections and the use of broad-spectrum antibiotics, increased medical costs, decreased quality of life, and increased mortality [1, 2]. Granulocyte-colony-stimulating factor (G-CSF), granulocyte–macrophage colony-stimulating factor (GM-CSF), and pegylated G-CSF are effective at reducing risk and duration of neutropenia-related negative events [3, 4]. Despite guidelines for the administration of these agents, their use in a clinical setting is inconsistent. The majority of studies that examined patterns of care report on Peg-/G-CSF use [5–8]. The purpose of this study was to (1) review the published literature on variations in patterns of care of G-CSF use and to identify overuse, underuse, and misuse rates and; (2) to identify patient and physician factors associated with overuse, underuse, and misuse.

## Neutropenia

Neutropenia is marked by abnormally low levels of white blood cells due to chemotherapy or other factors such as marrow infiltration from cancer, which ultimately predisposes patients to potentially life-threatening infections [9]. As noted by Bennett, chemotherapy is associated with a reduction in the efficacy of gut mucosa to serve as a barrier against microbial invasion leading to an increased risk for infection [10]. FN is the most serious manifestation of neutropenia and is defined as an absolute neutrophil count <0.5 × 10^9^/L accompanied by fever. In the United States, neutropenia-related hospitalization is estimated to occur in 34.2 cases per 1000 chemotherapy-treated patients, translating to approximately 60,000 cases every year [9]. The typical mortality rate associated with episodes of FN in various studies has ranged from 5% to 13.7%, although the risk may approach or exceed 50% in high-risk populations, based on age, comorbidity, disease characteristics, and myelotoxicity of the chemotherapy regimen [2, 11, 12]. Reported inpatient cost estimates are significant, ranging, on average, from $10,000 to $30,000 per neutropenia-related hospitalization [2, 13]. In addition, the management of FN in an outpatient setting may account for up to half of the total supportive care costs and approximately one-fifth of the total supportive care costs in an inpatient setting [14]. Neutropenia is also the primary dose-limiting cause for delays and reductions in relative dose-intensity, potentially compromising patient outcomes, including overall survival and complete response rates [15–17].

## Effectiveness of G-CSF

G-CSFs are biological growth factors that support the proliferation, differentiation, and activation of granulocytes. The United States and European guidelines support the routine use of G-CSF as primary prophylaxis (e.g., administered after the first cycle of chemotherapy) in patients receiving chemotherapy regimens where the risk of FN is >20% [1, 18–20]. A number of meta-analytic studies of randomized controlled trials have reported that the use of G-CSF for primary prophylaxis is associated with a shorter duration of neutropenia, shorter hospitalization, reduced risk of FN, and lower mortality rate due to infection [3, 4, 21]. However, in another meta-analysis of 148 studies, a significant reduction in infection-related mortality was not found although a significant reduction in infection was reported [22].

As a secondary prophylaxis (patients experienced neutropenia in a previous cycle and were given G-CSF prophylactically in a subsequent cycle), G-CSF reduces: time for neutrophil recovery, the incidence of FN, hospitalization, and the administration of broad-spectrum antibiotics [23]. While secondary prophylaxis is beneficial, studies comparing primary to secondary tend to support the use of the former over the latter [24]. Although not part of the recommended guidelines, G-CSF is often delayed until patients develop neutropenia or until FN has developed, and it is utilized as a therapeutic treatment for the established condition [8]. There is some evidence that using G-CSF is moderately effective in treating neutropenia and FN as an adjunctive to antibiotics [25].

It should be noted that despite its effectiveness, G-CSF use is not without some adverse events. The most frequently patient-reported adverse event is mild-to-moderate bone pain [26]. In addition, a in one study while the absolute risk was low, breast cancer patients treated with G-CSF were significantly more likely (hazard rate ratio = 2.14) to develop myelodysplastic syndrome or acute myeloid leukemia than patients not receiving G-CSF [27]. Rupturing of the spleen is also a rare but serious side effect of G-CSF [28].

## Overuse, Underuse, and Misuse of G-CSF

As mentioned earlier, United States and European guidelines [18–20] suggest that G-CSF to be used as primary prophylaxis after chemotherapy when the risk of FN is >20%. These guidelines do not recommend G-CSF for the therapeutic treatment of SN or FN [19]. However, in clinical practice, the G-CSF guidelines are often not consistently followed (see Table 1). Early evidence regarding inconsistency in adherence to G-CSF guidelines was reported first by Bennett and colleagues [6] over a decade ago. More recent physician surveys show similar patterns. For example, in a survey of more than 1200 members of the American Society of Clinical Oncology (ASCO), nearly a third of physicians reported using G-CSF prophylactically in patients at low risk for FN (<20%), and 48% indicated that they use G-CSF as an adjunct to antibiotics to treat FN [19]. More recently, a number of studies have report that the inconsistency of G-CSF administration is characterized by underutilization in high-risk patients and overutilization in patients at low risk [5, 7, 29].

**Table 1:** Summary of findings of underuse, overuse, and misuse of G-CSF

Reference	Overuse, underuse, or misuse	Major finding
Freifield et al. [19]	Overuse, underuse	Nearly a third of physicians reported using G-CSF prophylactically in patients at low risk for FN (<20%), and 48% indicated that they use G-CSF as an adjunct to antibiotics to treat FN
Wright et al. [8]	Overuse, underuse	62.1% of low-risk patients and 65.9% of high-risk patients received G-CSF to treat FN
Ramsey et al. [5]	Overuse, underuse	50% of high-risk patients received G-CSF; 21% of cancer patients at little or no risk received a G-CSF
Barron et al. [30]	Underuse	G-CSF prophylaxis was frequently used less often than antibiotic prophylaxis
Potosky et al. [7]	Underuse, misuse	17% of high-risk, 18% of intermediate-risk, and 10% of low-risk (<10%) patients received prophylactic G-CSF. In most cases, the use of G-CSF was therapeutic or reactive to preexisting FN
Waters et al. [29]	Overuse	46% of prophylactic G-pegylated CSF dosages were classified as not needed in patients undergoing low- or intermediate-risk cancer regimen

A recent examination of over 25,000 cancer patients admitted to the hospital for FN found no difference between the percentage of low-risk patients (62.1%) and high-risk patients (65.9%) receiving a G-CSF to treat (e.g., therapeutic use) their existing FN [8]. Another study found that 50% of cancer patients (e.g., breast, colorectal, or non–small-cell lung cancer) at high risk for FN received G-CSF as primary prophylaxis, while up to 21% of cancer patients at little or no risk for FN received a G-CSF [5]. A large cohort of high-risk patients for SN and FN in one U.S. hospital showed that G-CSF prophylaxis was frequently used less often than antibiotic prophylaxis even though FN was a common event, patients had lengthy hospital stays and high in-patient mortality rates, and the associated costs were high [30].

In a population-based, observational, multiregional cohort study of lung and colorectal cancer patients by Potosky and colleagues, only 17% of patients undergoing chemotherapy regimens at high risk (≥20%) for FN received prophylactic G-CSF [7]. In comparison, 18% of patients with intermediate risk (10–20%) for FN and 10% of patients with a low risk (<10%) were administered a G-CSF. Moreover, in most cases, the use of G-CSF was therapeutic or reactive to preexisting FN. In fact, the authors reported that 96% of all G-CSF administration fell outside the current guidelines. In another study highlighting overuse, Waters and colleagues identified patients administered pegylated G-CSF prophylactically while undergoing low- or intermediate-risk cancer regimen [29]. Thirty-seven percent of these patients had no risk factors for FN and 20% had only one risk factor. In fact, the authors reported that 46% of G-CSF dosages were classified as not needed (e.g., overutilized) resulting in excess healthcare costs of $712,264 in 1 year.

## Factors Associated with Overuse, Underuse, and Misuse of G-CSF

Given the effectiveness of G-CSF when used properly, it is important to identify the factors that lead to the inconsistency between guideline recommendations and actual clinical use of G-CSF. The current review has identified a number of potential factors that can be either classified as factors associated with the physician or the patient. Electronic databases (PubMed, PsycINFO, and Cochrane, ASCO abstract) were searched to identify studies published up to January 2014 using combinations of the following search terms “G-CSF,” “G-CSF,” “guidelines,” “predictors,” “patient (physician) characteristics,” “underutilization,” and “overutilization.”

### Physician factors

We found that the use of G-CSF can vary greatly not only between different cancer practices but between physicians within the same practice [31]. For example, the electronic treatment records from 10 oncology practices within the same management group revealed that in one practice, as few as 4% of patient treatment regimens included G-CSF, while in another practice G-CSF was used in 27% of regimens. The same study also found that within a single practice, the use of G-CSF varied from no use by one physician to use in 44% by another physician. These findings highlight the possibility that differences in the use of G-CSF could be due to factors associated with the treating physicians.

#### Physicians and healthcare setting

One of the most salient factors for the use of G-CSF is the setting in which the patient receives treatment. In two early surveys of ASCO members, doctors in HMOs or academic settings were less likely to use G-CSF as primary or secondary prophylaxis than physicians who practiced in fee-for-service settings [7, 32, 33]. In a survey assessing over 600 gynecological oncologists' attitudes toward G-CSF use, physicians in private practice were significantly more likely to have administered G-CSF as prophylaxis than physicians in an academic setting [34]. In a recent study of over 25,000 cancer patients hospitalized for FN, patients treated at teaching hospitals were less likely to receive a G-CSF to treat their FN than those treated in nonteaching hospitals [8].

Taken together, these studies suggest that physicians in an academic setting use G-CSF less than physicians in a fee-for-service setting. However, when taking into consideration how G-CSF is used, the differentiation is more nuanced. Prophylactic use of G-CSF may be underutilized by physicians in an academic setting and more often used by physicians in a fee-for-service setting. However, using G-CSF to treat FN occurs less frequently in academic settings compared to a fee-for-service setting. One possibility for this difference may be due to issues of reimbursement, which is less of a concern for physicians in fee-for-service than in an academic setting. In fact, Bennett and colleagues [6] found that a third of physicians surveyed said that they preferred not to use G-CSF if reimbursement was unlikely. Therefore, in a fee-for-service setting, where reimbursement is less of an issue, G-CSF may be more often used prophylactically.

Further evidence for the role of compensation was found by Potosky and colleagues, who reported that patients in HMO plans, where physician compensation is less tied to the drug and services administered, were less likely to receive a prophylactic G-CSF than patients in a non-HMO [7]. The authors reported that the percentage of breast cancer patients in high-risk chemotherapy regimens receiving G-CSF as primary prophylaxis was greater in patients with commercial insurance (33.4%) than in patients with Medicare (17.8%) or Medicaid (24.4%) even though preauthorization is not required by Medicare [7].

#### Physician knowledge/experience

In a 1999 study, physicians with higher hematology training, and with a higher number of patients with hematologic malignancies (more exposure to this population), were more likely to use G-CSF as primary prophylaxis [33]. Patients treated at hospitals that experience a high volume of patients with FN are also more likely to follow guidelines by treating patients with FN with antibiotics rather than G-CSFs [8].

### Patient factors

The guidelines for prophylactic use of G-CSF take into consideration a number of patient factors. Factors leading to a higher risk of FN and more likely use of prophylactic G-CSF includes being female, being over the age of 65, having advanced disease, or presenting with serious comorbidities [18, 19]. However, related studies have found that these factors are not consistently related to G-CSF use—either as prophylaxis or to treat FN.

#### Race

Members of racial and ethnic minorities do not always have the same access to or receive the same level of health care quality as nonminority patients [35]. There is some evidence of similar disparity driving differential G-CSF usage among different racial/ethnic groups in United States. For instance, a study using nationwide, population-based Medicare claims data found that African American women were significantly less likely to receive prophylactic G-CSF than women of any other race or ethnicity during treatment for breast cancer [36]. Instead, they are more likely to receive a G-CSF to treat FN which is counter to current guidelines [8].

#### Comorbidity

Although a severe comorbidity score is associated with an increased likelihood of G-CSF use either prophylactically or reactively in patients undergoing intermediate- or low-risk chemotherapy regimens [7], a study among older breast cancer patients with a comorbidity score of 3 or greater showed less likelihood of receiving a G-CSF as primary prophylaxis [36]. The same study also showed the geographic variations in the usage patterns. Finally, use of G-CSF to treat neutropenia has been shown to be higher in hospitalized patients with pneumonia or those admitted to the intensive care unit [8].

#### Geographic factors

Du and colleagues found that the use of G-CSF varied widely based on geographic region [36]. For example, they pointed out that only 10.6% of patients in Seattle received a G-CSF compared with 22.9% of patients in Atlanta. Meanwhile, compared with patients diagnosed in Seattle, patients in three metropolitan areas in California were more than twice as likely to receive a G-CSF. Similarly, in the recent study by Wright and colleagues, patients in the Midwest were less likely to receive G-CSF to treat FN compared to patients on the east coast, while patients in the West were more likely to receive a G-CSF to treat FN [8]. Differences in G-CSF use have also been noted between patients living in an urban versus a rural setting, with patients living in the former receiving G-CSF more frequently than the latter [27].

#### Patient beliefs/attitudes

Few studies have assessed patients' attitudes or knowledge of neutropenia and G-CSF use. One recent discrete choice survey of almost 300 cancer patients found that these patients chose G-CSF options with the lowest out-of-pocket expenses, the fewest injections, and lowest risk of disruption to chemotherapy schedule and infection requiring hospitalization most frequently [37]. In particular, out-of-pocket expenses and the risk reduction to disruption of chemotherapy schedule were most important to those surveyed patients. Patients reported that they were willing to pay $1076 per cycle to reduce the risk from high to low in delaying the chemotherapy schedule and $884 per cycle out-of-pocket expenses to lower the risk of hospitalization due to infection from 24% (high) to 7% (low).

#### Cancer type

Another factor that may impact the primary prophylaxis of G-CSF is the cancer type of the patient. In a retrospective database study of patients that received G-CSF as a primary prophylaxis, breast cancer was the most common tumor type, followed by non-Hodgkin's lymphoma and lung cancer [38]. Results from an observational study of community-based oncology practices revealed that primary prophylaxis with a G-CSF was most common in breast cancer, followed by lung cancer and then non-Hodgkin's lymphoma [39]. In a study of VA patients, the most common cancer in patients receiving G-CSF was non-Hodgkin's lymphoma (70.2%), followed by lung cancer (54.9%), and finally prostate cancer (32%) and colorectal cancer (32.8%). These U.S.-based data are similar to what Canada, Europe, and Australia found in a prospective observational study of routine clinical practices in [40]. Specifically, G-CSF primary prophylaxis was highest in breast cancer patients (55%), followed by small-cell lung cancer patients (32%). Twenty percent of ovarian and NSCLC patients received G-CSF as primary prophylaxis. Approximately 20% of patients in each tumor group received G-CSF as secondary prophylaxis.

#### Chemotherapy regimen

Ramsey and colleagues [5] examined the likelihood of receiving G-CSF as primary prophylaxis as a function of cancer type and chemotherapy dose regimen (high, intermediate, and low risk for FN). Among patients receiving high-risk chemotherapy regimens, patients with breast cancer were significantly more likely to receive G-CSF as primary prophylaxis than patients with non–small-cell lung carcinoma (NSCLC). Among patients undergoing intermediate-risk chemotherapy regimens, patients with breast cancer were significantly more likely to receive G-CSF as primary prophylaxis than patients with NSCLC, and patients with colorectal cancer were significantly less likely to receive G-CSF as primary prophylaxis. Among patients undergoing low-risk chemotherapy regimens, patients with breast cancer and colorectal cancer were less likely to receive G-CSF as primary prophylaxis than patients with NSCLC.

In another study, patients undergoing an intermediate-risk cancer regimen were more likely to have received G-CSF either as primary prophylaxis, as secondary prophylaxis or to treat FN than patients undergoing a low- or high-risk regimen [7]. Similar results were also found in a non-U.S. observational study in Spain where primary prophylaxis with a G-CSF was much more frequent in the high-risk subgroup (70.9% vs. 39.0% of patients at moderate risk). Patients receiving R-CHOP-21 were significantly less likely to receive a G-CSF than patients in an alternative dose-dense regimen [41].

#### Disease severity

Use of G-CSF has also been found to vary depending on the stage of the disease. For instance, in breast cancer patients, only 11% of patients with stage I received a G-CSF compared with 24% of patients with stage III and 17% of patients with stage IV [36]. This study also found that patients with a larger tumor were more likely to be administered a G-CSF; however, the authors did not control for chemotherapy regimen. Similar results were also reported by Rajan and colleagues [42]. Specifically, a larger proportion of breast cancer patients in stage III received a prophylactic G-CSF than those who were not. Furthermore, patients who received a prophylactic G-CSF had a larger tumor size and greater node positivity. In another study, G-CSF-treated patients had a significantly worse prognosis than nontreated patients [43].

## Conclusion

Despite the guidelines, many patients are receiving G-CSF when risks of FN are low and many are not receiving G-CSF when risks are high (>20%). In practice, many regimens have <20% risk and should not receive primary prophylaxis. Furthermore, many clinicians are using G-CSFs reactively to treat FN which is counter to current guidelines. As identified in this review, the reasons for the inconsistent prescribing patterns G-CSF are multifactorial and include differences among physician experience and training, practice setting (fee-for service vs. research hospitals) reimbursement, and the geographical location of care. As well as factors inherent to the patient or disease including race, geographic location of care, comorbidity, disease severity, chemotherapy regimen, and patients' beliefs and attitudes toward G-CSF.

Physician training and experience are associated with their adherence to guidelines. Specifically, Cabana and colleagues found that a lack of awareness and familiarity of guidelines were two of the most common barriers to physicians proper use of clinical guidelines [44]. Both of which are barriers that can be overcome, at least partially, by increased exposure to or education about the guidelines. In fact, one previous study has already shown that peer consultation leads to more appropriate use of G-CSF [45]. Specifically physicians in 22 community oncology practices covering approximately 97,000 Medicare HMO members participated in an educational intervention where the prophylactic use of G-CSF for a patient at low risk for FN was reviewed by a board-certified hematologist/oncologist. If the use of GCSF was deemed clinically, unwarranted the reviewing physician would explain to the attending physician why the use of GCSF in this low-risk patient was not necessary and provided published data from clinical trials as well as the guidelines. The attending physician made the final treatment decision after receiving the feedback. Before the peer-review process, the proportional use of GCSF in low-risk patients was 67.5%. Over the course of 1 year, this steadily decreased to 38.4%. There were no neutropenic-related episodes reported in low-risk patients that had the GCSF withheld after consultation.

This review also highlighted the importance of the hospital setting in which clinicians are working which may be a proxy for issues involving reimbursement. Specifically, prophylactic GCSF may be underutilized by physicians in an academic setting and more often used by physicians in nonacademic hospitals. As first suggested highlighted by Bennett and colleagues [6], this difference may be due to issues of reimbursement, which is less of a concern for physicians in nonacademic as opposed to an academic setting. Therefore, in a fee-for-service setting, where reimbursement is less of an issue, G-CSF may be more often used prophylactically. Additional research is needed to determine if the likelihood of reimbursement is also related to overutilization of G-CSF.

With respect to the patient factors, there is some initial evidence of racial disparity in G-CSF and additional research is needed to determine the breadth of race differences and whether they are confounded by cancer type. Furthermore, it is clear that cancer-type, severity of the disease, and chemotherapy regimen are each a factor that is associated with the differential use of G-CSF prophylaxis. Additional research is needed to more clearly understand these effects separately and in conjunction with one another given their confounding impact. Finally, additional research is needed to more closely examine patients' attitudes toward and knowledge of neutropenia and G-CSF. Insuring that patients have a better understanding of neutropenia's impact and the potential benefits (and risks) of G-CSF could lead to more appropriate use of G-CSF.
